# In Hypertrophic Cardiomyopathy Reduction of Relative Resting Myocardial Blood Flow Is Related to Late Enhancement, T2-Signal and LV Wall Thickness

**DOI:** 10.1371/journal.pone.0041974

**Published:** 2012-07-30

**Authors:** Katja Hueper, Antonia Zapf, Jan Skrok, Aurelio Pinheiro, Thomas A. Goldstein, Jie Zheng, Stefan L. Zimmerman, Ihab R. Kamel, Roselle Abraham, Frank Wacker, David A. Bluemke, Theodore Abraham, Jens Vogel-Claussen

**Affiliations:** 1 Department of Radiology, Johns Hopkins University School of Medicine, Baltimore, Maryland, United States of America; 2 Institute for Radiology, Hannover Medical School, Hannover, Germany; 3 Institute for Biometry, Hannover Medical School, Hannover, Germany; 4 Division of Cardiology, Johns Hopkins University School of Medicine, Baltimore, Maryland, United States of America; 5 Department of Electrical Engineering, Stanford University, Stanford, California, United States of America; 6 Mallinckrodt Institute of Radiology, Washington University School of Medicine, St. Louis, Missouri, United States of America; 7 National Institutes of Health, Radiology and Imaging Sciences, Bethesda, Maryland, United States of America; Ochsner Health System, United States of America

## Abstract

**Objectives:**

To quantify resting myocardial blood flow (MBF) in the left ventricular (LV) wall of HCM patients and to determine the relationship to important parameters of disease: LV wall thickness, late gadolinium enhancement (LGE), T2-signal abnormalities (dark and bright signal), LV outflow tract obstruction and age.

**Materials and Methods:**

Seventy patients with proven HCM underwent cardiac MRI. Absolute and relative resting MBF were calculated from cardiac perfusion MRI by using the Fermi function model. The relationship between relative MBF and LV wall thickness, T2-signal abnormalities (T2 dark and T2 bright signal), LGE, age and LV outflow gradient as determined by echocardiography was determined using simple and multiple linear regression analysis. Categories of reduced and elevated perfusion in relation to non- or mildly affected reference segments were defined, and T2-signal characteristics and extent as well as pattern of LGE were examined. Statistical testing included linear and logistic regression analysis, unpaired t-test, odds ratios, and Fisher’s exact test.

**Results:**

804 segments in 70 patients were included in the analysis. In a simple linear regression model LV wall thickness (p<0.001), extent of LGE (p<0.001), presence of edema, defined as focal T2 bright signal (p<0.001), T2 dark signal (p<0.001) and age (p = 0.032) correlated inversely with relative resting MBF. The LV outflow gradient did not show any effect on resting perfusion (p = 0.901). Multiple linear regression analysis revealed that LGE (p<0.001), edema (p = 0.026) and T2 dark signal (p = 0.019) were independent predictors of relative resting MBF. Segments with reduced resting perfusion demonstrated different LGE patterns compared to segments with elevated resting perfusion.

**Conclusion:**

In HCM resting MBF is significantly reduced depending on LV wall thickness, extent of LGE, focal T2 signal abnormalities and age. Furthermore, different patterns of perfusion in HCM patients have been defined, which may represent different stages of disease.

## Introduction

Hypertrophic cardiomyopathy (HCM) is a complex and relatively common genetic disorder with a prevalence of 0.2%. It is characterized by left ventricular (LV) hypertrophy in the absence of any other cardiac or systemic disease. HCM is heterogeneous in terms of various gene mutations, histopathology, presentation, clinical course and prognosis [1], [2], [3], [4]. Therefore, it is important to define subgroups that are at risk for adverse cardiac events, to allow personalized risk-adjusted treatment. Several risk factors have been identified, including non-sustained ventricular tachycardia, family history of HCM and sudden cardiac death, syncope, low blood pressure in response to exercise, LV hypertrophy ≥30 mm [5], [6], LV outflow tract obstruction [7] and extensive myocardial delayed enhancement (LGE) on magnetic resonance imaging (MRI) [8], [9].

Myocardial perfusion abnormalities are common in HCM patients and seem important for pathophysiology and prognosis [10]. They may be related to abnormal intramyocardial coronary arteries [11], inadequate capillary density in relation to increased myocardial mass or impairment of LV relaxation and may lead to myocardial ischemia and scarring. Nuclear medicine studies show that focal perfusion defects both at rest and during exercise are associated with arrhythmia, cardiac arrest and syncope in HCM patients [12], [13], [14], [15]. A higher degree of microvascular dysfunction has been shown to be an independent predictor for worse prognosis and death in HCM patients [16].

Local differences in myocardial blood flow (MBF) at rest and their relationship to tissue characteristics as evaluated by LGE and T2-weighted imaging have not yet been examined in detail. This is especially important as signal characteristics of a single sequence are not specific for certain pathologies in HCM: For example LGE in HCM patients may not only represent fibrosis, but also myocardial disarray, inflammation, and necrosis [17]. High signal intensity on T2-weighted images indicates edema and can be due to inflammation or ischemia [18], whereas dark signal on T2-weighted images indicates chronic fibrosis [19]. Combining LGE and T2-weighted MRI with perfusion imaging may be helpful to further characterize the complex histopathology and stage of lesions in HCM patients.

Therefore, the purpose of this study was to quantify resting LV MBF by MRI and to determine its relationship to important parameters of disease such as LV wall thickness, LGE, T2-signal changes, LV outflow tract obstruction and age.

## Methods

### Ethics Statement

This prospective, Health Insurance Portability and Accountability Act (HIPAA)-compliant study was approved by the Johns Hopkins Medicine institutional review board (Baltimore, MD, USA). Written informed consent was obtained from all participants.

### Study Population

Seventy patients with HCM were recruited mostly during their initial visit at our tertiary referral center and underwent cardiac MRI between November 2007 and May 2011. Patients with MRI contra-indications such as cardiac pacemaker and implantable cardioverter defibrillator (ICD) were excluded. Diagnosis of HCM was based on the presence of LV hypertrophy (end-diastolic LV wall thickness ≥15 mm) not originating from other causes [20]. The LV outflow gradient at rest was measured by Doppler echocardiography. Echocardiography and MRI were conducted at the same visit usually on the same day.

### MRI Protocol

All cardiovascular MRI examinations were performed on a 1.5 T MRI system (Avanto; Siemens Health Care, Erlangen, Germany) using a 6-channel array surface coil and a spine coil. Cine MRI was acquired in the short-axis view using a retrospective, electrocardiographically gated, balanced steady-state free precession (SSFP) sequence: Repetition time (TR)/ echo time (TE) 2.9/ 1.2 ms, flip angle 76°, matrix 256×154, slice thickness 8 mm, reconstructed cardiac phases 30. In order to assess myocardial T2-signal abnormalities a spectral attenuated inversion recovery (SPAIR) T2-weighted dark blood turbo spin echo sequence was applied in the short-axis view: 1400/ 76 ms TR/ TE, echo train length 13, matrix 256×186, slice thickness 8 mm. For perfusion imaging a bolus of gadopentetate dimeglumine (Bayer Schering Pharma, Berlin, Germany) was injected at a dose of 0.04 mmol/kg bodyweight, which was followed by a 20 ml saline flush; both were injected at a rate of 5 ml/s. Images were obtained using a saturation preparation SSFP sequence: TR/ TE 2.4/ 1.0 ms, inversion delay 180 ms, flip angle 40°, parallel acquisition acceleration factor 2 (generalized autocalibrating partially parallel acquisitions, GRAPPA), matrix 192×134, slice thickness 8 mm [21], [22]. Two short-axis slices at the basal and mid-ventricular level or at the mid-ventricular and apical level for apical forms of HCM were acquired during one heartbeat, resulting in a temporal resolution of one R-R interval. Short-axis segmented inversion-recovery gradient-echo turbo fast low angle shot (FLASH) images were obtained ten minutes after injection of an additional 0.16 mmol/kg gadopentetate dimeglumine dose (total dose  =  0.2 mmol/kg) using the following parameters: TR/ TE 2.9/ 3.3 ms, flip angle 25°, TI as determined from TI scout, matrix 256×156, slice thickness 8 mm.

Imaging planes and positions of cine, T2-weighted and LGE MRI were matched to the slice location of the cardiac perfusion sequence to allow direct comparison of all parameters within the same myocardial region. Cine and LGE sequences covered the entire left ventricle, but only the matching slices were used for comparison with myocardial perfusion.

### MRI Analysis

**Figure 1:**
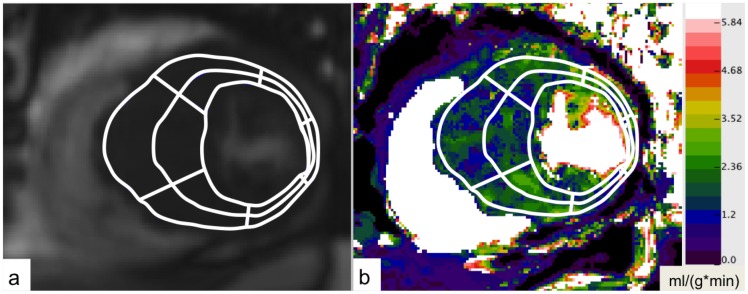
Pixel-by-pixel calculation of quantitative MBF maps. Quantitative MBF map in the short axis view of an HCM patient. MBF values are given in ml/(g*min). A region of interest (ROI) was drawn, outlining the LV walls. The ROI was divided into 6 segments per slice with further subdivision in epi- and endocardial half.

Quantitative analysis of cine and perfusion MRI were performed by one reader (XX 2 years of cardiac MRI experience), who was blinded to clinical history and echocardiographic findings. In order to determine local differences of myocardial perfusion, LV wall thickness, LGE and T2-signal, the LV myocardium was divided into 6 segments per slice in each of the acquired short-axis slice according to the recommendation of the American Heart Association [23]. Each segment was further subdivided into an endocardial and an epicardial subsegment (Fig. 1).

Using dedicated cardiac software (MASS 7.2, Medis, Leiden, The Netherlands) LV mass index, LV end-diastolic and end-systolic volume index and ejection fraction were determined from short-axis cine MRI. The end-diastolic LV wall thickness was measured in each segment, matching the cardiac perfusion segments.

**Figure 2:**
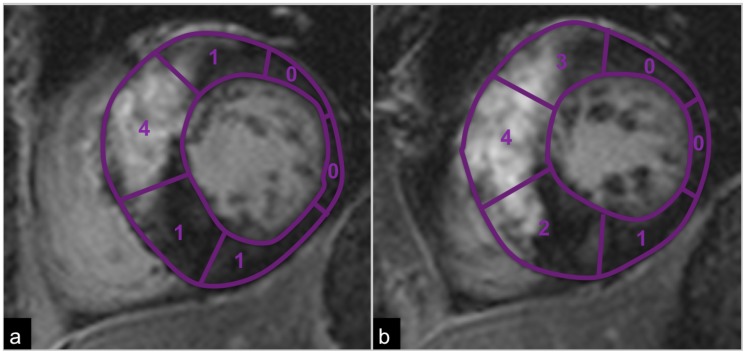
Semiquantitative evaluation of LGE. LGE was scored semiquantitatively in each segment based on the percentage area of LGE. Depicted is an example for LGE scoring: 0 = no LGE, 1 = 1–25%, 2 = 26–50%, 3 = 51–75% and 4 = 76–100% LGE.

T2-signal changes and LGE were scored visually in corresponding slices and segments by consensus of two experienced readers (YY 9 years of cardiac MRI experience and XX). Edema, defined as focally increased T2-signal when compared to remote T2-signal, was scored as absent  =  0 or present  =  1 for each myocardial segment. Similarly, T2 dark signal (chronic fibrosis), defined as focally reduced T2-signal when compared to remote T2-signal, was recorded as absent  =  0 or present  =  1. The extent of LGE within each segment was scored visually from 0 to 4 based on the percentage area with hyperenhancement: 0  =  no LGE, 1  =  1–25%, 2 = 26–50%, 3 = 51–75% and 4 = 76–100% LGE (Fig. 2).

For absolute quantification of MBF, perfusion maps were generated on a pixel-by-pixel basis using the Fermi function model and dedicated perfusion software as previously described [24], [25], [26], [27]. Perfusion images were motion corrected and denoised [28]. The arterial input function was obtained by placing a region of interest (ROI) in the LV cavity. On both slices of the short-axis perfusion MRI an ROI was drawn, outlining the LV walls. Myocardial segments were defined as described above and ROIs were copied to perfusion maps (Fig. 1). For each segment and each epi- and endocardial half mean MBF was calculated and corrected for heart rate and blood pressure by dividing the absolute MBF by the rate-pressure product and multiplying the quotient by 10,000 [29], [30].

**Figure 3:**
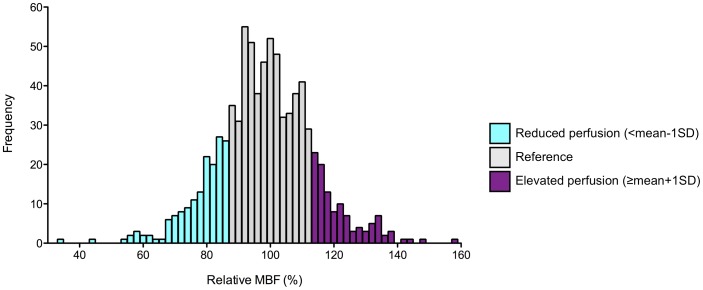
Frequency and range of relative resting MBF. Frequency and range of relative resting MBF of all segments (n = 804 segments, n = 70 patients). Segments with reduced perfusion in relation to reference segments <mean -1 SD (86.75%) are highlighted blue, segments with elevated perfusion >mean +1 SD (113.25%) purple. Segments within 1 SD of reference segments are defined as reference (grey).

To further assess regional differences in MBF, relative perfusion values were calculated. In each patient, we first defined reference segments as non- or mildly affected segments with myocardial thickness <20 mm and LGE score 0–1 in the segment as well as the respective epi- and endocardial subsegments. These thresholds were chosen because some patients did not have segments without LGE and myocardial wall thickness <15 mm. The mean MBF of reference segments in one patient was calculated and relative perfusion for all segments in one patient was then calculated in relation to this mean value. In addition, mean relative MBF of all reference segments of all 70 patients (per definition 100%) and the standard deviation (SD) were determined and three categories of relative perfusion were defined: reduced perfusion <mean -1 SD (86.75%), elevated perfusion ≥mean+1 SD (113.25%) and perfusion within one SD of reference segments (86.75–113.25%), referred to as reference (Fig. 3).

### Statistical Analysis

Statistical analysis was performed using SAS 9.2 (SAS Institute Inc., Cary, NC, USA). Values are expressed as mean and 95% confidence interval [CI]. P <0.05 was considered to indicate significance.

In order to characterize the association of LGE with T2 signal changes and LV wall thickness a logistic regression with repeated measures was performed. The presence of LGE (score 1–4) vs. no LGE (score 0) was defined as response variable; LV wall thickness, edema and T2 dark signal as independent factors. In a linear regression model with repeated measures the relationship of relative resting MBF with LV wall thickness, LGE score, edema score, T2 dark score, LV outflow gradient at rest (determined by Doppler echocardiography) and age was determined. All statistically significant parameters (p<0.05) were then evaluated together in a multiple linear regression model. Although the distribution of relative resting MBF was slightly skewed to the left, we regarded the data as approximately normally distributed owing to the rather large sample size.

As relative resting MBF was heterogeneous even in segments with LGE (range 33.5–147.2%), a subgroup analysis including all segments with LGE (score 1–4) was performed. Within this subgroup segments with reduced or elevated perfusion in combination with LGE (n = 78 and n = 24 segments, respectively) were evaluated for the presence of LV wall thickness ≥20 mm, focally elevated and decreased T2-signal and the pattern of LGE. The frequency of those parameters in reduced and elevated perfusion segments of this subgroup was compared to the frequency of parameters in reference segments of the subgroup using Fisher’s exact test; odds ratios and relative risks were calculated. The respective extent of LGE in elevated and reduced perfusion segments in this subgroup was compared using an unpaired t-test. It should be noticed that the subgroup analysis was performed descriptively on a per segment basis, and only descriptive p-values are given.

## Results

### Study Population

In 70 HCM patients (mean age 51.7 years, CI [47.7, 55.8]) 804/840 segments were included in the analysis. Patient characteristics are given in Table 1. 36 segments had to be excluded completely due to motion artifacts. In addition 24 segments had severe motion artifacts in T2-weighted MRI and were excluded from T2 analysis only.

**Table 1:** Characteristics of HCM patients .

Age, y	51.7 [47.7, 55.8]
Male gender, %	80
Heart rate, bpm	64.4 [62.2, 66.7]
Blood pressure, mmHg	132 [128, 136]
LV outflow gradient at rest, mmHg	27.2 [19.8, 34,5]
LV EDV index, ml/m^2^	62.7 [59.0, 66.5]
LV ESV index, ml/m^2^	12.3 [11.2, 13.4]
LV ejection fraction, %	80.1 [78.6, 81.6]
LV mass index, g/m^2^	98.5 [91.3, 105.7]
Patients with LGE (score 1–4), n (%)	54 (77%)
Segments with LGE (score 1–4), n (%)	258 (32%)
Patients with LV wall thickness ≥20 mm, n (%)	36 (51%)
Segments with LV wall thickness ≥20 mm, n (%)	102 (13%)
Patients with T2 bright signal (edema), n (%)	25 (36%)
Segments with T2 bright signal (edema), n (%)	120 (15%)
Patients with T2 dark signal, n (%)	18 (26%)
Segments with T2 dark signal, n (%)	40 (5%)
Normalized MBF at rest, ml*min^−1^*g^−1^*(mmHg*bpm/10^4^) ^−1^	1.54 [1.43, 1.65]

Results for n = 70 patients with diagnosis of HCM and a total of 804 segments (12 segments per patient) are given as mean [95% confidence interval (CI)]. EDV, end-diastolic volume; ESV, end-systolic volume; LV, left-ventricular; normalized MBF, myocardial blood flow corrected for the rate-pressure product; LGE, late gadolinium enhancement.

All morphological phenotypes of HCM were included: 45 septal, 9 mid-wall, 7 apical and 9 diffuse types. LGE was detected in 258 segments (32%) within 54 patients. In a logistic regression model the odds for the presence of LGE were 1.25 (CI [1.18; 1.31], p<0.001) for each millimeter increase in end-diastolic LV wall thickness. The odds for the presence of LGE were 16.31 (CI [7.96; 33.43], p<0.001) in segments with focal T2 bright signal and 14.02 (CI [5.40; 36.42], p<0.001) in segments with focal T2 dark signal (Table 2).

**Table 2:** Results of simple logistic regression with LGE as the outcome.

Parameter	Comparison	Odds Ratio	95% CI	p-value
Myocardial thickness	global	1.25	1.18; 1.31	<0.001
Edema	1 vs. 0	16.31	8.00; 33.43	<0.001
T2 dark signal	1 vs. 0	14.02	5.40; 36.42	<0.001

**Table 3:** Results of simple and multiple regression analysis with relative MBF as the outcome.

Parameter	Comparison	Simple linear regression			Multiple linear regression		
		Regressioncoefficient (β)	95% CI	p-value	Regressioncoefficient (β)	95% CI	p-value
LV wallthickness	global	**−**0.3	**−**0.5; **−**0.1	<0.001	**−**0.1	**−**0.1; 0.3	0.431
LGE	global			<0.001			<0.001
	1 vs. 0	**−**2.9	**−**5.6; **−**0.2	0.037	**−**1.8	**−**4.8; 1.1	0.220
	2 vs. 0	**−**5.9	**−**10.1; **−**1.7	0.006	**−**3.9	**−**8.7; 0.9	0.108
	3 vs. 0	**−**12.9	**−**17.7; **−**8.0	<0.001	**−**8.9	**−**14.6; **−**3.2	0.002
	4 vs. 0	**−**23.2	**−**31.2; **−**15.1	<0.001	**−**19.5	**−**28.4; **−**10.6	<0.001
Edema	1 vs. 0	**−**8.3	**−**5.2; **−**11.3	<0.001	**−**4.0	**−**0.5; **−**7.5	0.026
T2 dark signal	1 vs. 0	**−**12.3	**−**7.2; **−**17.4	<0.001	**−**6.5	**−**1.2; **−**11.8	0.019
Age	global	**−**0.07	**−**0.14; **−**0.01	0.032	**−**0.05	**−**0.11; 0.01	0.108
LV outflowtract gradient	global	0	**−**0.03; 0.04	0.901	-	-	-

Results of regression analysis with relative MBF as the outcome. LV wall thickness, age and gradient are continuous variables; LGE (score 0–4), edema (score 0–1) and T2 dark signal (score 0–1) are categorical variables.

### Resting Myocardial Perfusion

Absolute resting MBF was not significantly different between segments with varying severities of myocardial wall thickening or LGE. Also there were no significant differences between absolute epi- and endocardial resting MBF.

In a simple linear regression model for repeated measurements LV wall thickness (p<0.001), LGE score (p<0.001), edema (p<0.001), T2 dark signal (p<0.001) and age (p = 0.032) but not LV outflow gradient (p = 0.901) showed a significant effect when correlated with relative resting perfusion (Table 3). For example, an increase of LV wall thickness of 1 mm was associated with an average reduction of relative myocardial perfusion of 0.3%. An increase of ten years in age was related to an average reduction of relative MBF of 1%. Multiple linear regression analysis including LV wall thickness, LGE score, edema score, T2 dark score and age as parameters revealed that LGE (p<0.001), edema (p = 0.026) and T2 dark signal (p = 0.019) were independent predictors for a reduction of relative resting perfusion (Table 3). In this model, only high extent of LGE (score 3 and 4) but not low extent of LGE (score 1 and 2) was an independent predictor for reduced resting perfusion. For example, on average relative MBF in a segment with LGE score 4 was 19.5% lower than in a segment without LGE (score 0). In segments with edema (score 1) the average relative MBF was 4.0% lower compared to segments without edema (score 0). In segments with T2 dark signal (score 1) the average relative MBF was 6.5 % lower than in segments without T2 dark signal (score 0).

### Subgroup Analysis of Segments with LGE (score 1–4) in Categories of Reduced and Elevated Perfusion

**Figure 4:**
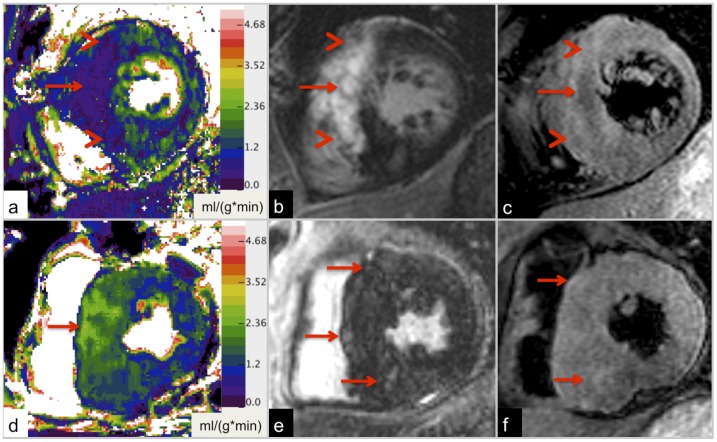
Examples of different perfusion patterns in HCM. 21-year-old patient with non-obstructive HCM (LV outflow gradient 7 mmHg). Large area of intense, well-defined LGE in the anterior septum and the anterior wall (b) with corresponding hypo-perfusion (relative MBF  = 33.5%; a). T2-weighted imaging depicts low signal in the central area of the lesion, suggesting macroscopic, chronic fibrosis/ scar tissue (red arrow), and adjacent high signal, indicating edema (arrow head, c). Maximum LV wall thickness is 31 mm. Findings of a 46-year-old HCM patient with an LV outflow gradient of 11 mmHg are given in d-f. Resting MBF of the interventricular septum is focally increased to 145% (d). In this area patchy and diffuse LGE with relatively low signal intensity (e) and patchy edema on T2-weighted images (f) are visible (red arrows). Maximum LV wall thickness is 33 mm.

LGE (score 1–4) was present in 49.4% (78/158) of segments with reduced perfusion (<mean -1 SD) and in 22.2% (24/108) of segments with elevated perfusion (>mean +1 SD). In reduced perfusion segments LGE was more frequently accompanied by T2 bright signal (edema), T2 dark signal (chronic fibrosis) and increased LV wall thickness ≥20 mm compared to reference segments. In elevated perfusion segments LGE was only more frequently accompanied by increased LV wall thickness compared to reference segments (Table 4). The extent of LGE was higher in reduced perfusion segments (mean LGE score, 2.11, CI [1.88; 2.35]) than in elevated perfusion segments in this subgroup (LGE score 1.54, CI [1.26; 1.82], p = 0.014). The pattern of LGE in reduced perfusion segments (Fig. 4a-c) was mainly characterized by high signal intensities and was well circumscribed (54/78 segments). In contrast, regions of elevated perfusion were associated with a diffuse and patchy LGE of low to intermediate signal intensity (23/24 segments), but not frequently with foci of T2 dark signal (3/23, Fig. 4d-e).

**Table 4:** Comparison of frequency of LGE and frequency of LV wall thickening ≥20 mm and T2 signal changes accompanied by LGE in segments of different perfusion categories.

	Reference segments	Reduced perfusion segments				Elevated perfusion segments			
Parameter	Frequency	Frequency	Relativerisk	Oddsratio	p-value	Frequency	Relativerisk	Oddsratio	p-value
LGE	156/538 (29%)	78/158 (49%)	1.70	2.39	<0.001	24/108 (22%)	0.77	0.70	0.160
LGE + LV wall ≥20 mm	38/156 (24%)	34/78 (44%)	1.79	2.40	0.004	11/24 (46%)	1.88	2.63	0.046
LGE + T2 bright area	44/146 (30%)	45/78 (58%)	1.91	3.16	<0.001	10/23 (44%)	1.44	1.78	0.232
LGE + T2 dark area	15/146 (10%)	22/78 (28%)	2.75	3.43	0.001	3/23 (13%)	1.27	1.31	ns

Comparison of the frequency of LGE (score 1–4) in segments with reduced and elevated perfusion to segments with perfusion values within one standard deviation of reference segments (reference segments). Similarly the frequencies of LGE accompanied with LV wall thickness ≥20 mm or accompanied with edema (T2 bright) and T2 dark signal were compared. Due to artifacts ten segments in the reference group and one segment in the elevated perfusion group were excluded from the T2 signal analysis. Relative risk, odds ratio and p-values are given for the comparison between reduced and elevated perfusion segments to reference segments.

LV, left-ventricular; LGE, late gadolinium enhancement; ns, not significant.

## Discussion

In this study focal differences of relative resting MBF in HCM patients were observed using MRI. Relative perfusion correlated inversely with LV wall thickness (p<0.001), extent of LGE (p<0.001), edema (p<0.001), T2 dark signal changes (p<0.001) and age (p = 0.032). The LV outflow gradient did not affect resting myocardial perfusion, which is consistent with previous studies [16].

Myocardial perfusion is an important parameter in HCM, because perfusion abnormalities may lead to ischemia and myocardial scarring and thus, increase the risk for adverse cardiac events such as arrhythmias and LV dysfunction. For instance, a reduction of myocardial perfusion reserve at hyperemia or during exercise, indicating microvascular dysfunction, has frequently been described in HCM patients [12], [13], [15], [31]. Cecchi et al. demonstrated its prognostic relevance in HCM: The degree of microvascular dysfunction was associated with worse prognosis and death [16]. Petersen et al. showed by cardiac perfusion MRI reduced MBF values at hyperemia in HCM, which were proportional to the degree of end-diastolic LV wall thickness [32]. Perfusion reserve was predominantly reduced in the endocardial layer and in segments with LGE.

In comparison, in our study alterations of resting perfusion could only be appreciated as relative changes, because absolute MBF was highly variable between patients. This is in agreement with previous studies demonstrating that absolute resting MBF is not different among segments with differing severities of myocardial wall thickening or LGE and between epi- and endocardial myocardium [32], [33]. Romero-Farina et al. showed in a myocardial perfusion SPECT study that perfusion defects at rest were associated with a higher prevalence of severe complications, indicating the prognostic relevance of resting myocardial perfusion [12]. In our study, extent of LGE and presence of T2-signal abnormalities (T2 bright and T2 dark signal) independently predicted reduced resting perfusion. Relative MBF also decreased with increasing LV wall thickness, which was, however, mainly due to the higher prevalence of LGE in segments with increased LV wall thickness. The Odds Ratio for presence of LGE was 1.25 for each millimeter increase of end-diastolic wall thickness, which is consistent with observations by Petersen et al. [32].

It has been suggested that LGE in HCM is mainly attributed to macroscopic fibrosis and focal increase of collagen [8], [34], [35]. Accordingly, LGE was associated with relatively reduced resting MBF in our study. However, even within LGE segments high relative perfusion values up to 147.2% were observed. In the subgroup of segments with LGE (score 1–4), the extent of LGE was higher in reduced perfusion segments than in elevated perfusion segments (Fig. 4a-c); in reduced perfusion segments LGE was often characterized by high signal intensities and was relatively well circumscribed. Furthermore, LGE in reduced perfusion segments was more frequently accompanied by edema (T2 bright signal) and T2 dark signal than in reference segments (Fig. 4a-c). Thus, LGE in segments with low relative perfusion might represent more advanced stages of myocardial involvement in HCM with a predominance of macroscopic, confluent fibrosis [36]. The T2 dark signal within areas of LGE may represent a high amount of chronic/old fibrotic tissue with very low resting perfusion similar to the decreased T2-signal in scars after chronic myocardial infarction [19].

In contrast, regions of elevated perfusion (Fig. 4d-e) were associated with diffuse and patchy LGE of low to intermediate signal intensity. T2 signal abnormalities were not more frequently observed than in reference segments. With regard to histopathological features of HCM, areas of focal increase of relative resting MBF might represent early disease with predominance of abnormal intramural coronary arteries before macroscopic fibrosis occurs [11]. Thus, resting perfusion might be increased as a result of compensatory reaction mechanisms, inflammation or due to a lack of auto-regulation of intramural arteries. Interestingly, the pattern of LGE in hyperperfused segments is similar to the diffuse LGE-pattern Moon et al. described [8]. He found that patients with this LGE-pattern have more risk factors for sudden cardiac death.

Furthermore, the fact that LGE was also accompanied by increased perfusion in some segments in our study supports the hypothesis of Knaapen et al., that LGE in HCM patients not only represents macroscopic fibrosis but is also related to myocardial disarray, microscopic fibrosis, abnormal intramural coronary arteries and inflammation [17]. They compared areas of LGE with the perfusable tissue index determined by ^15^O-PET imaging, which is known to decrease with the extent of fibrosis in chronic myocardial infarction [37]. In areas of LGE in HCM patients, however, the perfusable tissue index was not reduced when compared to controls and the extent of LGE even positively correlated with this index. Perfusion MRI at rest, therefore, might add additional information to LGE and T2-weighted imaging about the underlying activity and stage of disease in HCM and may be of prognostic relevance.

A limitation of the present study is the calculation of relative perfusion values. In HCM, alterations of myocardial perfusion have been demonstrated even in segments without morphological changes [33]. Therefore, the segments chosen for calculation of relative MBF may be an imperfect reference. Furthermore, perfusion imaging did not cover continuously the entire left ventricle. In addition, as MRI and echocardiography were not conducted at the same time, varying preloading conditions may have influenced the measurement of LV outflow gradients and myocardial perfusion [38], [39]. Also, no myocardial biopsies were obtained to directly correlate the underlying histopathology with the described resting perfusion patterns. T2 and LGE images were scored visually by consensus reading, volume of affected tissue was not calculated. However, especially in HCM patients even “remote” myocardium may have abnormal signal, which can make quantification challenging. Novel T1 and T2 mapping MRI techniques could be more objective and solve this dilemma.

In conclusion, quantitative mapping of MBF allows for visualization and quantification of focal changes of resting myocardial perfusion in patients with HCM. The extent of LGE and the presence edema and focal T2 dark signal changes were independent predictors of segments with reduced relative resting MBF. Different myocardial resting perfusion patterns have been defined, which may represent different stages of disease. Further research needs to determine if the described resting perfusion patterns are predictors for clinical outcome in HCM patients.
